# A Deep Insight into Different Acidic Additives as Doping Agents for Enhancing Proton Conductivity on Polybenzimidazole Membranes

**DOI:** 10.3390/polym12061374

**Published:** 2020-06-18

**Authors:** Jorge Escorihuela, Abel García-Bernabé, Vicente Compañ

**Affiliations:** 1 Departamento de Química Orgánica, Facultad de Farmacia, Universitat de València, Av. Vicent Andrés Estellés s/n, 46100 Burjassot, Valencia, Spain; 2 Departamento de Termodinámica Aplicada, Escuela Técnica Superior de Ingeniería Industrial, Universitat Politècnica de València, Camino de Vera s/n, 46022 Valencia, Spain; agarciab@ter.upv.es

**Keywords:** fuel cells, proton conductivity, electrochemical impedance spectroscopy, polymer, polybenzimidazole, proton exchange membrane, phosphoric acid, phytic acid, phosphotungstic acid

## Abstract

The use of phosphoric acid doped polybenzimidazole (PBI) membranes for fuel cell applications has been extensively studied in the past decades. In this article, we present a systematic study of the physicochemical properties and proton conductivity of PBI membranes doped with the commonly used phosphoric acid at different concentrations (0.1, 1, and 14 M), and with other alternative acids such as phytic acid (0.075 M) and phosphotungstic acid (HPW, 0.1 M). The use of these three acids was reflected in the formation of channels in the polymeric network as observed by cross-section SEM images. The acid doping enhanced proton conductivity of PBI membranes and, after doping, these conducting materials maintained their mechanical properties and thermal stability for their application as proton exchange membrane fuel cells, capable of operating at intermediate or high temperatures. Under doping with similar acidic concentrations, membranes with phytic acid displayed a superior conducting behavior when compared to doping with phosphoric acid or phosphotungstic acid.

## 1. Introduction

**Figure 1:**
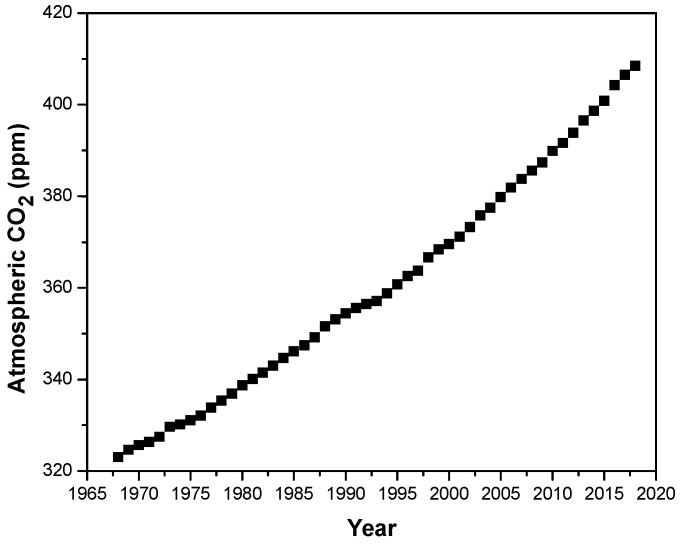
Atmospheric CO_2_ concentration (ppm) in the past 25 years.

Carbon dioxide concentration in the atmosphere has reached worrying values above 400 ppm in the last months (Figure 1) [1]. These alarming values have focused researchers’ objectives towards the search for novel and sustainable energy systems to fulfil the world demand [2]. Fuel cells are electrochemical devices that convert chemical energy into electrical energy in an efficient way [3]. Among the different types of fuel cell, proton exchange membrane fuel cells (PEMFCs), which use an ion exchange polymer film as the electrolyte, have received special attention due to their low operating temperature and quick start-up [4,5]. The main component of a PEMFC is the proton exchange membrane (PEM), which is responsible for proton transport between the anode and the cathode [6]. In this regard, the synthesis of novel polymer electrolyte materials with high chemical, thermal and mechanical stability combined with elevated conductivity, has absorbed the market over the past few decades [7,8,9]. Among the wide array of polymers used for this purpose, Nafion is the most commonly used PEM material and possesses high proton conductivity as well as excellent chemical stability at low temperatures [10]; however, its conductivity drops at temperatures above 90 °C, hampering its use as a high temperature PEMFC [11]. The advantages of working at temperatures above 120 °C include a remarkable reduction in CO poisoning of the catalyst [12], improvement of diffusion rates and redox reactions [13], and simplification in water and heat management [14]. In the quest for novel polymeric electrolytes for energy applications operating at high temperatures [15], several polymeric families have been developed in order to replace the widely used perfluorinated polymers [16,17,18]. Among these novel polymeric materials, polybenzimidazole (PBI) derivatives have emerged as potential candidates due to their higher thermal and mechanical stability [19].

PBI (poly[2,2′-(m-phenylen)-5,5′-bisbenzimidazole]) is a heterocyclic, thermoplastic, basic, and hydrophilic synthetic polymer with a very high glass transition temperature (425–436 °C) [20]. Since its development in 1961 by H. Vogel and C.S. Marvel [21], PBI has been used by NASA in the Apollo missions as part of the astronauts’ clothing, but it was not until 1995 that it was used in fuel cells by Wainright et al. [22]. Despite possessing high and thermal chemical stability, combined with good mechanical stability, the proton conductivity of PBI membranes is relatively low without chemical modification of the PBI structure or the introduction of fillers into the PBI matrix. In this regard, the most widely used strategy to increase proton conductivity in PBI-based membranes is acid doping. In particular, the use of phosphoric acid (PA) has been established as a standard approach [23,24,25,26,27], despite drawbacks such as acid leaching and membrane degradation [28,29]. Throughout the past decades, a variety of alternatives to PA have been used in an attempt to improve the physicochemical properties and performance of PBI membranes, such as the incorporation of inorganic fillers [30,31], metallic salts [32,33], carbon-based materials [34,35], zeolitic imidazolate frameworks (ZIFs) [36,37,38], and ionic liquids (ILs), among others [39,40,41,42].

**Figure 2:**
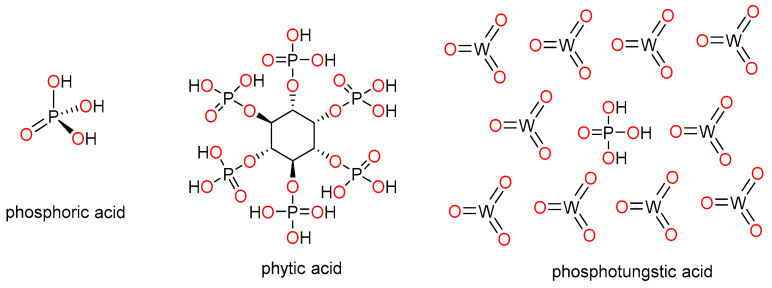
Chemical structures of phosphoric acid (PA), phytic acid, and phosphotungstic acid (HPW).

As mentioned above, phosphoric acid (PA) is by far the most extensively used approach to enhancing proton conductivity of PBI-based membranes. After acid doping, conductivity can be enhanced by several orders of magnitude, reaching values in the range of 0.1–0.2 S·cm^−1^, which are comparable to Nafion membranes operating at 80 °C under high hydration conditions. However, acid leaching studies and stability tests after a few operating cycles are generally omitted in these studies. Among other doping acids used to increase proton conductivity in polymer electrolyte membranes, phytic acid (myoinositol hexakisphosphate) and phosphotungstic acid (HPW) are among the most prominent and have emerged as more sustainable alternatives to replace phosphoric acid (Figure 2). Phytic acid is a phosphorus-containing organic acid that is present in plants, especially in seeds and fiber. This acid has been used as a doping agent for polymer electrolyte membranes based on Nafion, yielding excellent proton conductivities [43,44]. On the other hand, HPW, an heteropoly acid with molecular formula H_3_P_4_W_12_O_40_, has also been efficiently applied as a proton carrier in proton exchange membrane fuel cells [45,46,47,48].

Continuing our ongoing work towards developing novel proton exchange membranes based on polybenzimidazole for high temperature PEMFCs, we present a systematic study of physicochemical properties and proton conductivity of PBI membranes doped with phosphoric acid at different concentrations (0.1, 1, and 14 M) and other alternative acids, such as phytic acid (0.075 M) and HPW (0.1 M). The use of these three acids was reflected in the formation of channels in the polymeric network as observed by cross-section SEM images. These membranes exhibited improved proton conductivity compared to undoped PBI membranes. We also studied other properties of the PBI-doped membranes, including their phosphoric acid uptake, thermal stability, and mechanical strength. Finally, we also calculated the proton diffusion coefficient (*D*) using the Nernst–Einstein equation.

## 2. Materials and Methods

### 2.1. Materials

PBI (purity >99.95%, molecular formula: (C_20_H_12_N_4_)_n_, MW 51,000,) was purchased from Danish Power Systems. Lithium chloride (LiCl), *N*,*N*-dimethylacetamide (DMAc) 99.8%, phosphoric acid (extra pure, 85% solution in water), phytic acid solution (50% (*w/w*) in H_2_O), sodium hydrogen phosphate (Na_2_HPO_4_), and sodium tungstate dihydrate (Na_2_WO_4_·2H_2_O) were purchased from Sigma-Aldrich (Aldrich, Madrid, Spain). The phosphotungstic acid solution was prepared using Na_2_WO_4_·2H_2_O and Na_2_HPO_4_ as precursors, by mixing 1.10 g of Na_2_HPO_4_ and 5.95 g of Na_2_WO_4_·2H_2_O in 30 mL of deionized water at 50 °C.

### 2.2. Membrane Preparation.

Initially, a DMAc solution containing 0.1 wt.% of LiCl (used as a stabilizer) was prepared by dissolving 100 mg of LiCl in 100 mL of DMAc under vigorous stirring (1 h at room temperature) to give a homogeneous solution. Next, a 10 wt.% PBI solution was prepared by dissolving PBI powder (10 g) in the DMAc solution (90 g). The mixture was heated under reflux at 120 °C for 6 h. The final prepared solution had a viscosity of 0.5 Pa·s at 25 °C. Then, PBI membranes were prepared via the solution cast method. To this end, the PBI solution was cast onto a clean glass slide and dried under vacuum at 80 °C for 16 h and finally at 140 °C for 10 h. Membranes were washed with distilled water at 80 °C in order to remove residual solvent (DMAc) and LiCl. Traces of the solvent were finally removed by drying at 160 °C for 16 h. The membrane thicknesses prior to acid doping varied between 100 and 120 μm. Finally, membranes were doped with the corresponding acid by immersion in the acidic solution for 48 h at room temperature.

### 2.3. Membrane Characterization

Scanning electron microscopy (SEM) images were obtained using a field emission scanning electron microscope (FE-SEM) model Ultra 55 (Zeiss) operating at 5 kV with energy-dispersive X-ray (EDX) spectroscopy. Attenuated total reflection Fourier transform infrared (ATR-FTIR) spectra of the membranes were obtained using a Jasco FTIR spectrometer FT/IR-6200 Series (Jasco) with a 4 cm^−1^ resolution between 4000 and 600 cm^−1^. Thermogravimetric analysis (TGA) was performed on a TGA Q50 thermogravimetric analyzer TGA Q50 (Waters) under nitrogen atmosphere (60 mL·min^−1^) from 30 to 800 °C using a heating rate of 10 °C·min^−1^. The acid uptake (AU) of the membrane was calculated by the following equation: AU (%) = [(W_wet_ − W_dry_)/W_dry_] × 100; where W_wet_ and W_dry_ refer to the membrane’s weight after its immersion in the acid solution for at least 48 h at room temperature and the membrane’s weight after drying at 110 °C for at least 24 h, respectively. The thickness uptake (TU) of the membrane was calculated by the following equation: TU (%) = [(T_wet_ − T_dry_)/W_dry_] × 100; where T_wet_ and T_dry_ refer to the membrane’s thickness weight after drying at 110 °C for 24 h, respectively. The tensile properties of the membranes were calculated from stress–strain curves obtained using a precision universal/tensile tester (Shimadzu AGS-X) at a crosshead rate of 10 mm·min^−1^ at room temperature. To this end, membranes (five samples of each membrane) with a thickness around 100 μm were cut into strips of 30 mm × 6 mm and tested. Proton conductivity measurements (in the transversal direction) were performed using a broadband dielectric spectrometer (Novocontrol Technologies, Montabaur, Germany) integrated with an SR 830 lock-in amplifier with an alpha dielectric interface from 20 to 200 °C by electrochemical impedance spectroscopy (EIS) in the frequency interval of 0.1 Hz to 10 MHz, applying a 0.1 V signal amplitude. Initially, the temperature was gradually raised from 20 to 120 °C in steps of 20 °C and the dielectric spectra were collected at each step. During the measurements, the temperature was isothermally controlled using a nitrogen jet (Quatro from Novocontrol, Montabaur, Germany) with a temperature error of 0.1 °C during every single sweep in frequency.

## 3. Results and Discussion

### 3.1. Membrane Preparation and Characterization

**Figure 3:**
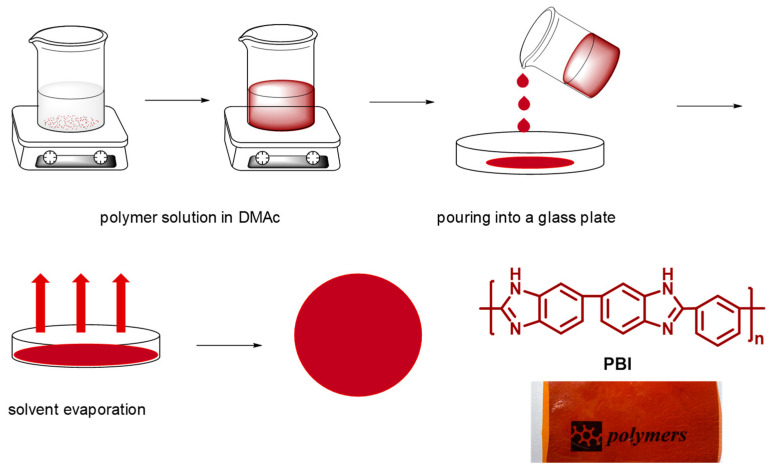
Schematic representation of polybenzimidazole (PBI) membrane preparation by the casting method.

PBI membranes were prepared by the casting method (Figure 3). For this purpose, a 10 wt.% PBI solution was prepared using a DMAc solution containing 0.1 wt.% of LiCl (used as a stabilizer). Shortly, PBI powder was completely dissolved in the DMAc solution, and then the homogeneous solution was cast onto a clean glass plate and heated in a vacuum oven at 80 °C for 16 h to completely remove the DMAc, and finally heated at 140 °C for 10 h. Using this methodology, transparent membranes with a uniform thickness of around 100 μm were obtained.

Next, PBI membranes were doped with phosphoric acid (0.1, 1, and 14 M), phytic acid (0.075 M), and HPW (0.1 M). Specifically, the acid-doped membranes were obtained by soaking the membranes in the corresponding acid solution at room temperature for 2 days to ensure complete saturation in the membrane. After this time, the membranes were removed, wiped down, and dried in a vacuum oven at 120 °C for 48 h to obtain a constant weight. The acid doping was confirmed by FTIR by the presence of a broad band centered at 1000 cm^−1^ corresponding to phosphonate groups (see Figure S1). FTIR spectra of membranes doped with phosphoric and phytic acid, displayed this intense characteristic band; however, it was very low for the phosphotungstic acid membrane, indicating a low degree of doping of this heteropoly acid in the PBI–HPW membrane.

Acid uptake and swelling are critical parameters to be considered when studying polymeric membranes for PEM fuel cell applications [49]. In this regard, the performance of a membrane is generally evaluated according to its proton conductivity, which is strongly dependent on its water or acid content. In this regard, high proton conductivity is associated with high levels of acid uptake; at the same time, it is also a sign of low-dimensional stability, as acid modifies the polymer microstructure and mechanical properties. Table 1 shows the acid uptake (AU), swelling, and thickness uptake (TU) of the PBI membranes measured at room temperature after immersion of 48 h in deionized water (DIW), phosphoric acid at different concentrations (0.1, 1, and 14 M), and phytic acid and HPW, at 0.075 and 0.1 M, respectively. The acid doping level was calculated by measuring the weight changes between dry doped membranes (W_acid_) and dry undoped membranes (W_dry_), where M_acid_ and M_PBI_ represent the molecular weight of acid (97.99 for PA, 660.04 for phytic acid, and 2880.05 for HPW) and the repeating unit of PBI (308.34 for C_20_H_12_N_4_), respectively.

(1) $$\mathrm{acid}\;\mathrm{doping}\;\mathrm{level}=\frac{(W_{\mathrm{acid}}-W_{\mathrm{dry}})/M_{\mathrm{acid}}}{W_{\mathrm{dry}}/M_{\mathrm{PBI}}}$$

As mentioned above, the acid doping level is a fundamental parameter for proton transport and expresses the number of acid molecules per monomer unit in the polymeric network. Accordingly, the higher the acid doping level of the membrane, the higher its proton conductivity. As shown in Table 1, the acid doping level was dependent on acid concentration and, as expected, higher acid doping levels were obtained for the membrane doped using the highest concentration (PBI–PA 14 M). Accordingly, higher swelling and TU were also observed for higher acid concentrations. In contrast, low acid doping levels were obtained when using phytic acid and HPW as alternative acids to conventional phosphoric acid doping, as also observed in previous studies by Kawakami and coworkers [44]. In this regard, to have a fair comparison, acid doping levels need to be compared with PBI–PA 0.1 M, as phytic acid and HPW were used at concentrations of 0.075 and 0.1 M, respectively. Due to the low solubility of phytic acid in water, the concentration of phytic acid corresponds to the commercially available phytic acid solution (50% (*w/w*) in H_2_O).

**Figure 4:**
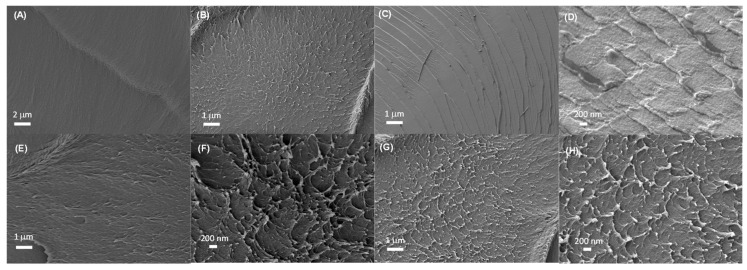
SEM images of cross-sections of PBI membranes after immersion in DIW (**A**,**B**), phosphoric acid 0.1 M (**C**,**D**), phytic acid 0.075 M (**E**,**F**), and HPW 0.1 M (**G**,**H**).

The internal microscopic morphologies of membranes were studied by SEM images. The SEM images of cryo-fractured cross-sections of the different PBI membranes are shown in Figure 4. The cross-section morphology of the undoped PBI membrane was dense and free of holes. However, after the addition of PA, the morphology of all membranes showed the formation of channels due to the presence of the acidic filler, reflected in the appearance of holes in the cross-section SEM images. After PA doping, the morphology of all membranes showed the formation of channels in the polymer network due to the presence of PA molecules, as observed in similar systems [50]. The doping with phytic acid and HPW also showed the presence of microstructures which might be involved in the conduction process. It could also be observed that the rough cross-section microscopic morphologies of PA-doped membranes resulted in a rough fracture cross-section attributed to the plasticizing effect of phosphoric acid.

**Figure 5:**
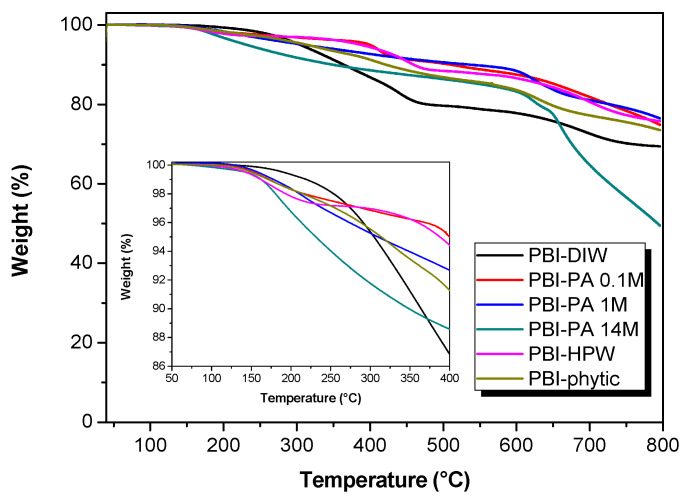
Thermogravimetric analysis of PBI membranes doped with different acids and concentrations. Inset: zoom at the 50–400 °C region.

One of the major properties to be considered in the development of proton exchange membrane for fuel cell applications is their thermal stability [51]. The thermal behavior of the membranes was studied via thermal analysis under N_2_ atmosphere with a 10 °C·min^−1^ heating rate. As shown in Figure 5, the thermal stability of PBI membranes was investigated by thermogravimetric analysis (TGA). For the PA-doped membranes, all the curves displayed a similar trend. The first degradation step was observed at 160 °C, which is attributed to the formation of pyrophosphoric acid through a condensation reaction of phosphoric acid. A second step was observed at about 600 °C and was associated to the degradation of the PBI main chain and the continuous dehydration of the pyrophosphoric acid to polyphosphoric acid. The results are similar to previously reported PBI membranes [46]. The weight loss curves for the PBI–phytic acid and PBI–HPW membranes presented a similar degradation trend. Furthermore, all the membranes showed similar T_d5%_ to the pristine PBI membrane. From the thermogravimetric analysis it can be concluded that the reported membranes possess an adequate thermal stability for their application as proton exchange membrane fuel cells for intermediate or high temperatures.

The study of mechanical properties of polymer electrolyte membranes is of the utmost importance for future application as PEM fuel cells [52]. In this regard, PEMs possessing excellent mechanical properties are highly demanded; however, high values of PA doping generally produce a decrease in the mechanical strength of PBI membranes, as PA molecules reduce the interaction between polymeric chains. The tensile properties of the acid-doped membranes were determined from stress–strain curves obtained with a universal testing machine at a crosshead rate of 10 mm·min^−1^ at room temperature. For that, rectangular samples (30 mm × 6 mm) with a thickness of 150 μm (five samples of each type of membrane) were tested and the average results are given in Table 2 with the corresponding standard deviation. For a better comparison, the Young′s modulus, tensile stress, and elongation at break values of the pure PBI dry membrane are also included. The undoped pristine PBI membrane had a Young’s modulus of 2.52 GPa, a tensile strength of 174 MPa, and an elongation at break of 18%. As observed, the immersion in water decreased both Young′s modulus and tensile stress. Meanwhile, the value of elongation at break increased. Phosphoric acid doping produced a similar effect, being more marked as the acid concentration increased, due to the plasticizing effect of phosphoric acid. The mechanical properties of the phosphoric acid-doped PBI membranes can be improved by lowering the acid doping level; however, the proton conductivity is dramatically reduced. This significant reduction of the PA-doped PBI membrane strength has been reported by other researchers in the literature [53,54]. PBI membranes doped with low concentrations of other acidic fillers, such as phytic acid and HPW, also displayed high values of elongation at break, in the range of 119–125%. The values are comparable with those of other reported PBI membranes [55,56,57,58]. It should be noted that all the composite membranes exhibited a tensile strength of above 2.0 MPa, which was strong enough for the fabrication of membrane electrode assemblies (MEAs) that could be evaluated in fuel cell performance tests [59,60].

### 3.2. Proton Conductivity

The analysis of proton conductivity is of the utmost importance for evaluation of a membrane to be considered as a membrane electrode assembly (MEA) in the manufacturing of proton exchange membrane fuel cells (PEMFCs). Among the different techniques generally used to measure proton conductivity in membranes, electrochemical impedance spectroscopy (EIS) has emerged as a powerful electrochemical technique to measure the proton conductivity of PEMFCs [61,62,63]. The through-plane conductivity of the PBI-doped membranes at different temperatures (from 20 to 200 °C) was determined by EIS using a blocking electrode configuration.

**Figure 6:**
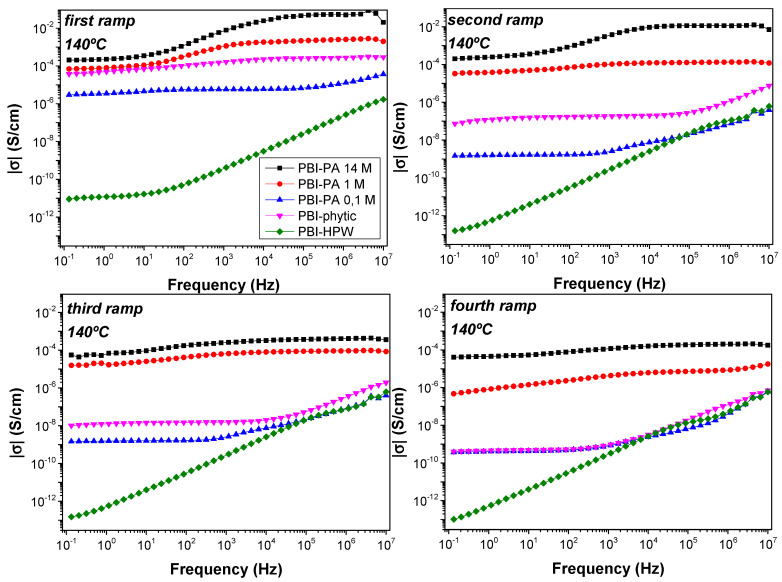
Bode diagrams at 140 °C for the acid-doped membranes: PBI–PA 0.1 M (▲), PBI–PA 1 M (●), PBI–PA 14 M (■),PBI–phytic acid (▼), and PBI–HPW (♦) measured in different cycles.

Generally, the proton conductivity in acid-doped membranes based on PBI are generally reported on a unique measurement at different temperatures. However, proton conductivity is not constant and generally drops after a few operating cycles. In order to evaluate the stability of proton conductivity in the acid-doped membranes, proton conductivity of the membranes was evaluated in four consecutive cycles. To this end, in the first ramp, measurements were performed in steps of 20 °C from 20 to 200 °C. Then, the sample was cooled down and a second ramp of measurements was applied in the temperature interval from 200 to 20 °C. This procedure was systematically repeated in the third (from 20 to 200 °C) and fourth ramp (200 to 20 °C) of measurements (see Figure S2). The dc-conductivity was obtained from the Bode diagram, which shows the real part of the conductivity (σ) as a function of the frequency. Ideally, the proton conductivity of the membrane can be obtained from the value of the conductivity in the region of high frequencies where the conductivity reaches a plateau [64]. Accordingly, the proton conductivity was obtained from the Bode plots in the different measurement cycles (Figure 6).

**Figure 7:**
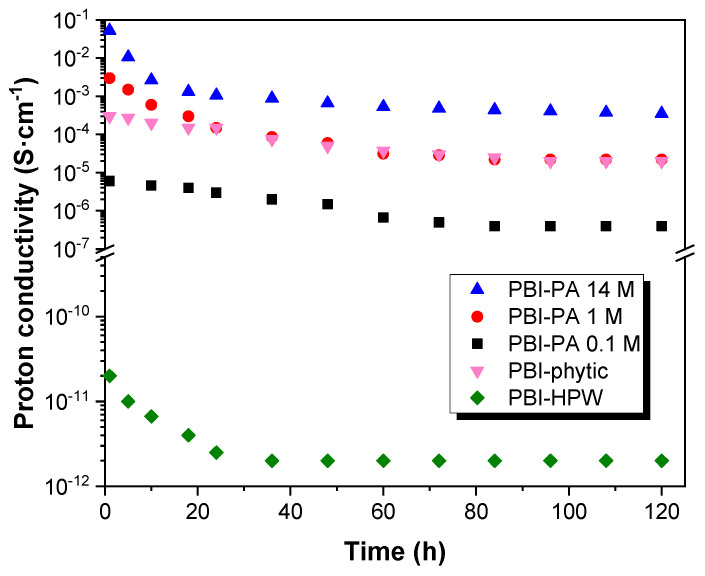
Long-term conductivity study at 160 °C for PBI–PA 0.1 M (■), PBI–PA 1 M (●), PBI–PA 14 M (▲), PBI–phytic acid (0.075 M) (▼), and PBI–HPW (0.1 M) (♦) membranes.

As shown in Figure 6, the values obtained for the dc-conductivity (σ) varied depending on the measurement cycle. Consequently, these results show that the conductivity of the membranes does not remain constant with temperature and time, which can be associated to the varying density of charge carrier into the membranes. In this regard, conductivity decreased along the different measurement cycles, changing about two orders of magnitude from the first to the fourth ramp in the case of PA-doped membranes PBI–PA 14 M and PBI–PA 1 M; around three orders of magnitude for the sample PBI–PA 0.1 M, and four orders of magnitude for the PBI–phytic acid membrane. This effect is attributed to the rapid loss of the free phosphoric acid molecules from the doped membranes. The leaching problems of acids from PBI membranes were evaluated in a long-term conductivity study of the membranes at 25 °C showing that a significant decrease in conductivity was observed after 24 h (Figure S3). This drawback was also observed in a long-term conductivity study at 10 °C (Figure 7), which is a usual operating temperature for high temperature (HT)-PEMFC membranes. As observed, and although PBI–PA 14 M membrane has a high proton conductivity, its value decreased around two orders of magnitude after 24 h, indicating an important acid leaching. Interestingly, PBI–phytic acid membrane has a lower leaching drawback and its conductivity at 160 °C was similar to that of PBI–PA 1 M membrane, contrary to what was observed at 25 °C, where PBI–PA 1 M membrane had a superior conductivity value. This result shows that phytic acid can be a promising candidate to be used at high temperatures. However, the use of other fillers such as ionic liquids and metal organic frameworks, among others, can help to more efficiently retain the acidic additive.

Considering the first cycle, the Bode diagrams in all temperature ranges (Figure S4) showed that the conductivity increased as the temperature increased. As expected, in the PA-doped membranes, the proton conduction depended on the concentration of phosphoric acid molecules. As described, the phosphoric acid molecules can interact with the imidazole ring in the PBI backbone, allowing protons to jump between the PBI network and promote proton transport through hydrogen bond formation and cleavage processes [65,66]. The proton conductivities at 40 °C were 5.6 × 10^−7^, 2.9 × 10^−4^, and 1.8 × 10^−2^ S·cm^−1^, while at 140 °C they were 5.8 × 10^−6^, 2.5 × 10^−3^, and 5.3 × 10^−2^ S·cm^−1^ for PBI–PA 0.1 M, PBI–PA 1 M, and PBI–PA 14 M, respectively. It is worth noting that phosphoric acid possesses a high intrinsic proton conductivity, which is mainly attributed to the presence of polarizable hydrogen bonds in a dense network [67]. Conductivities of PBI–phytic acid and PBI–HPW at the same temperatures were 3.2 × 10^−5^ and 4.1 × 10^−6^ S·cm^−1^, which decreased to 2.6 × 10^−4^ and 1.9 × 10^−11^ S·cm^−1^, respectively, at 140 °C. When comparing the conductivity of PBI–phytic acid and PBI–HPW membranes, the phytic acid analogue has a conductivity several orders of magnitude higher than the latter at all temperatures studied. Despite proton conductivities obtained with membranes doped with alternative acids such as phytic acid and HPW being different to those obtained with concentrated phosphoric acid (PBI–PA 14 M or PBI–PA 1 M), some interesting trends can be observed when compared to the sample with a similar concentration of acidic filler (PBI–PA 0.1 M). First, the PBI–HPW membrane displayed higher conductivity than PBI–PA 0.1 M, but lower than PBI–phytic acid membrane at low temperatures (below 80 °C). However, a strong decrease in proton conductivity was observed for the PBI–HPW membrane at 100 °C. A plausible explanation can be attributed to the evaporation of water molecules, which hampers proton transport under anhydrous conditions. Heteropoly acids such as HPW, generally exist in hydrated phases, in which the water molecules can form bridges between ionic clusters to facilitate proton mobility [68]. Secondly, the PBI–phytic acid membrane displayed a higher performance than PBI–PA 0.1 M membranes, both being in the same order of acid concentration. From these results, we can conclude that proton conductivity is several orders of magnitude higher for the membrane of PBI–phytic acid (2.6 × 10^−4^ S·cm^−1^ at 140 °C) when compared to PBI–HPW (1.9 × 10^−11^ S·cm^−1^), and a few orders of magnitude higher than the PBI membrane doped with phosphoric acid at 0.1 M (5.8 × 10^−6^ S·cm^−1^). These results may pave the way for the use of this natural acid obtained from plants with unique properties, such as a high phosphate groups content and good chemical and thermal stability [69].

**Figure 8:**
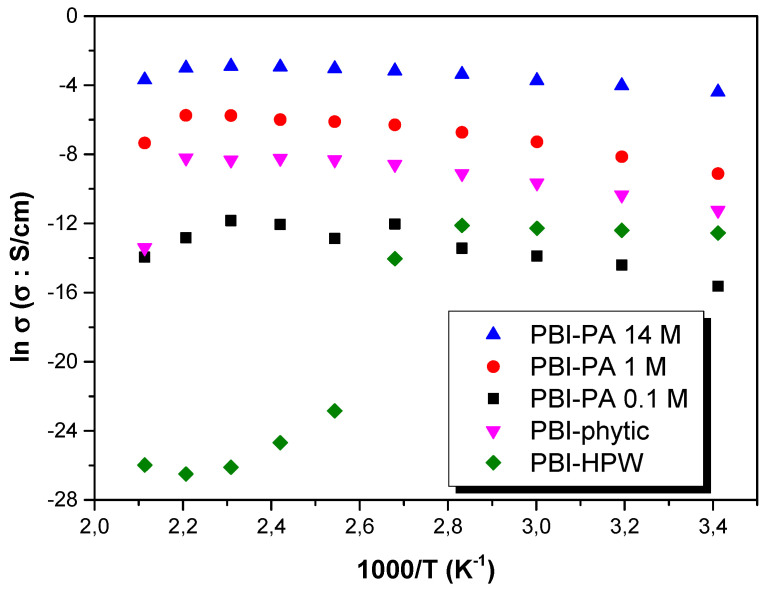
Temperature dependence of proton conductivity (σ) in all the range of temperatures for PBI–PA 0.1 M (■), PBI–PA 1 M (●), PBI–PA 14 M (▲), PBI–phytic acid (0.075 M) (▼), and PBI–HPW (0.1 M) (♦) membranes.

In order to further study the proton conduction mechanism of the membranes, the Arrhenius plots of all the membranes and their proton conduction activation energy values (E_act_) were analyzed. Figure 8 shows the tendency of the conductivity (σ) with temperature for all the membranes in the range of temperatures between 20 and 200 °C. As observed, proton conductivity increases for all membranes from 20 to 180 °C, following typical Arrhenius behavior. However, for the PBI–HPW membrane, a strong decrease in proton conductivity is observed at 100 °C. Despite HPW having one of the strongest acidities among the different heteropoly acids, its solubility in water is very limited and this drawback has limited its use in PEMFCs, as only a few reports based on PBI [70,71,72] and other polymeric matrices [73,74] have been described.

As observed, proton conductivity (*σ* in S·cm^−1^) followed typical Arrhenius behavior, where a linear tendency can be described in all the range of temperatures following the equation

(2) $$\ln\sigma =\ln\sigma_{0}-\frac{E_{act}}{RT}$$

where *σ_0_* is a pre-exponential factor (S·cm^−1^), *E_act_* is the activation energy (kJ⋅mol^−1^), and *R* is the ideal gas constant (8.314 J⋅K^−1^⋅mol^−1^). Using Equation (2), the activation energy was obtained from the slope of the linear fit for each sample. In this regard, the obtained values (see Table 3 for exact values) followed the trend: E_act_ (PBI–PA 14 M) ~12 < E_act_ (PBI–PA 1 M) ≈ E_act_ (PBI–phytic) = ~25 < E_act_ (PBI–PA 0.1 M) = ~28 < < E_act_ (PBI–HPW) = 31 kJ·mol^−1^. These results indicate that proton mobilities increased with the amount of phosphoric acid (i.e., the increase of PA concentration produces a decrease in activation energy which can be attributed to the increasing number of charge density of carriers (protons)). In other words, PA forms channels in the organic phase of porous PBI, as observed by SEM analysis of polymer morphology, facilitating the mobility and consequently, increasing proton conductivity.

Typically, proton conduction in polymeric membranes refers to the process of transport of hydrogen ions in one direction and can be rationalized according to two conduction pathways: the Grotthuss mechanism [75] and the vehicle mechanism [76]. In the Grotthuss mechanism, the proton transport is rationalized by means of the jump of protons in a hydrogen bond network composed by the different groups capable of forming hydrogen bonds, both from the PBI and acidic filler. On the other hand, the proton transport can be explained via the vehicle mechanism through the free phosphoric acid molecules or other acids present in the PBI matrix. The Grotthuss mechanism is characterized by a lower activation energy and according to the calculated activation energy (Table 3), the Grotthuss mechanism dominates the proton transport in acid-doped PBI membranes. Similar activation energies were obtained for PBI–PA 1 M, PBI–PA 0.1 M, and PBI–phytic acid membranes, but PBI–PA 14 M displayed a lower value as the PA concentration is much higher and therefore, the proton transport is more favored. This activation energy is similar to other reported PBI–PA-doped membranes [77,78] and that obtained for an 85% phosphoric acid solution [79].

**Figure 9:**
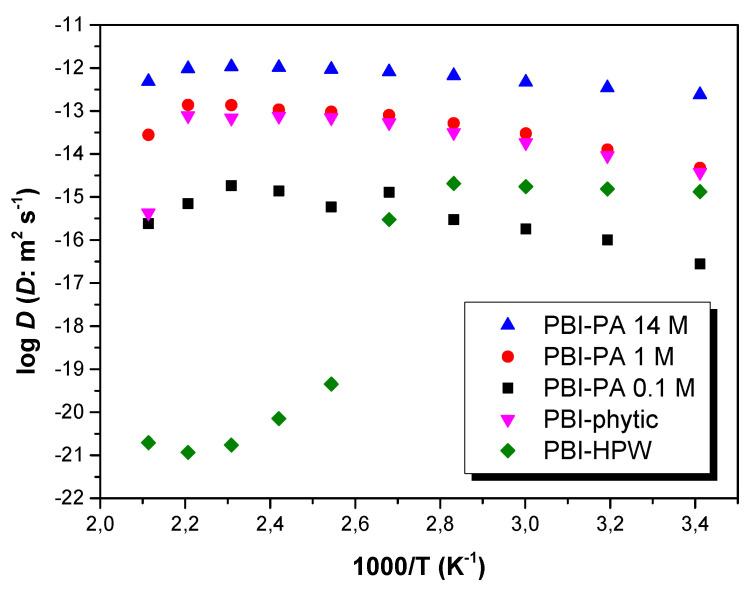
Temperature dependence of the diffusion coefficient (*D*) for PBI–PA 0.1 M (■), PBI–PA 1 M (●), PBI–PA 14 M (▲), PBI–phytic acid (0.075 M) (▼), and PBI–HPW (0.1 M) (♦) membranes.

Assuming that protons are the only available ions that can participate in the charge transport, the diffusivity (*D*) can be estimated from the conductivity (σ) which was previously determined with the Bode diagrams. According to this consideration, the ionic diffusivity can be estimated applying the Nernst–Einstein equation [80]

(3) $$D=\frac{\sigma RT}{F^{2}C_{+}}$$

where *C*_+_ is the concentration of ions in the membrane, σ the dc-conductivity, *R* is the gas constant, *F* is the Faraday constant, and *T* is the absolute temperature. Considering the stoichiometric amount of acid doping, the calculated density of protons was 5.8 × 10^−3^, 19.3 × 10^−3^, and 85.1 × 10^−3^ mol·cm^−3^, for PBI–PA 0.1 M, PBI–PA 1 M, and PBI–PA 14 M membranes, respectively. On the other hand, the calculated density of protons for the PBI–phytic acid (0.075 M) and PBI–HPW (0.1 M) membranes was 4.2 × 10^−3^ and 5.2 × 10^−3^ mol·cm^−3^, respectively. From these estimated values, the diffusion coefficient of the different membranes can be calculated from Equation (3). The calculated diffusion coefficients (*D*) at different temperatures are shown in Figure 9. As observed, the diffusion coefficient follows a similar behavior to conductivity from 20 to 180 °C (i.e., typical Arrhenius behavior). Similarly, for PBI–HPW membrane, a strong decrease is observed from 80 °C.

A comparison between the proton diffusion coefficient at 140 °C obtained from Equation (3), shows values of 1.4 × 10^−15^, 1.0 × 10^−13^, and 1.0 × 10^−12^ m^2^·s^−1^ for PBI–PA 0.1 M, PBI–PA 1 M, and PBI–PA 14 M, respectively. The calculated proton diffusion coefficients (*D*) increased, as expected, with higher acid concentration. In the case of PBI–phytic acid and PBI–HPW membranes containing acid concentrations of 0.075 and 0.1 M, respectively, diffusion coefficients of 1.4 × 10^−14^ and 1.0 × 10^−21^ m^2^·s^−1^ were obtained. As observed, for membranes with low acid doping (concentrations around 0.1 M), the proton diffusion coefficient for PBI–phytic acid was higher than that for PBI–PA 0.1 M and PBI–HPW membranes, as observed by its superior proton conduction, also reflected by the differences in the activation energies (Table 3).

Finally, a deep look at the variation of diffusion coefficients with temperatures shows that diffusion coefficients increase with temperature in agreement with the increase of proton conductivity. The values found in this work for diffusion coefficients are quite similar to other polymer electrolytes based on polyethylene oxide (PEO) containing Pr_4_N^+^I^-^ salts, whose values were around 1.8 × 10^−12^ m^2^·s^−1^ at temperatures below 100 °C [81]. On the other hand, the values found in this work are 10^4^ times higher than the diffusion coefficients of conductive ions obtained in copolymer of vinylidene cyanide and vinyl acetate determined from dielectric measurements using the model of Trukhan at temperatures of 195 °C, where the concentration of electrolytes present in the polymer was around 20 × 10^−3^ mol cm^−3^ [82]; this is quite similar to our concentrations of 5.8 × 10^−3^, 19.3 × 10^−3^, 85.1 × 10^−3^, 4.2 × 10^−3^, and 5.2 × 10^−3^ mol·cm^−3^ for PBI–PA 0.1 M, PBI–PA 1 M, PBI–PA 14 M, PBI–phytic acid (0.075 M), and PBI–HPW (0.1 M) membranes, respectively.

## 4. Conclusions

In conclusion, polybenzimidazole membranes containing different acids were prepared and their performance as HT-PEMFCs was evaluated through the analysis of proton conductivity, which was studied by EIS using a blocking electrode configuration. The use of these three acids was reflected in the formation of channels in the polymeric network as observed by cross-section SEM images. These doped materials maintained their mechanical properties and thermal stability for their application as proton exchange membrane fuel cells capable of operating at intermediate or high temperatures. The proton conductivity was strongly dependent on the measurement cycle and decreased along the different cycles. The acid doping increased proton conductivity of PBI membranes reaching values of 0.05 S·cm^−1^ at 140 °C for PBI–PA 14 M. Under low acid doping (concentrations around 0.1 M), membranes doped with phytic acid displayed a superior conducting behavior (2.6 × 10^−4^ S·cm^−1^) when compared to doping with phosphoric acid (5.8 × 10^−6^ S·cm^−1^) or phosphotungstic acid (1.9 × 10^−11^ S·cm^−1^). The results obtained with phytic acid doping may pave the way for the use of this natural acid in combination with other fillers as a sustainable alternative to the use of phosphoric acid and improve its retention in the polymeric membrane. Further applications of this natural acid with PBI membranes are currently under investigation.

## Figures and Tables

**Table 1:** Acid uptake (AU), swelling, and thickness uptake (TU) of the PBI membranes doped at room temperature under different conditions. DIW, deionized water.

Membrane	AU (%)	Swelling (%)	TU (%)	Acid Doping Level (%)
PBI–DIW	4 ± 1	5 ± 1	4 ± 1	----
PBI–PA 0.1 M	19 ± 1	9 ± 1	8 ± 1	0.60
PBI–PA 1 M	63 ± 3	17 ± 1	22 ± 2	1.98
PBI–PA 14 M	278 ± 5	63 ± 4	80 ± 3	8.74
PBI–phytic acid	21 ± 1	12 ± 1	10 ± 1	0.10
PBI–HPW	25 ± 1	15 ± 1	9 ± 1	0.03

**Table 2:** Mechanical properties of the PBI membranes doped under different conditions.

Membrane	Young’s Modulus (GPa)	Tensile Stress (MPa)	Elongation at Break (%)
PBI–dry	2.52 ± 0.17	174 ± 4	18 ± 2
PBI–DIW	2.13 ± 0.21	98 ± 6	66 ± 3
PBI–PA 0.1 M	1.58 ± 0.19	92 ± 5	89 ± 5
PBI–PA 1 M	1.22 ± 0.12	75 ± 4	108 ± 7
PBI–PA 14 M	0.11 ± 0.02	19 ± 2	189 ± 9
PBI–phytic acid	1.11 ± 0.15	71 ± 2	119 ± 5
PBI–HPW	1.67 ± 0.13	90 ± 4	125 ± 6

**Table 3:** Proton conductivities (σ) for all membranes under study at 20, 80, and 140 °C (obtained from the first measurement ramp) and calculated activation energies (E_act_).

Membrane	σ _20 °C_ (S·cm^−1^)	σ _80 °C_ (S·cm^−1^)	σ _140 °C_ (S·cm^−1^)	E_act_ (kJ·mol^−1^)
PBI–DIW	1.1 × 10^−14^	1.4 × 10^−13^	2.5 × 10^−12^	52.6 ± 2.1
PBI–PA 0.1 M	1.6 × 10^−6^	2.7 × 10^−6^	5.8 × 10^−6^	27.5 ± 3.8
PBI–PA 1 M	5.8 × 10^−4^	1.2 × 10^−3^	2.5 × 10^−3^	24.7 ± 3.2
PBI–PA 14 M	2.5 × 10^−2^	3.4 × 10^−2^	5.3 × 10^−2^	11.6 ± 0.7
PBI–phytic acid	1.3 × 10^−5^	1.1 × 10^−4^	2.6 × 10^−4^	25.1 ± 2.3
PBI–HPW	4.8 × 10^−6^	2.9 × 10^−6^	1.9 × 10^−11^	30.8 ± 2.8

## Supplementary Materials

The following are available online at https://www.mdpi.com/2073-4360/12/6/1374/s1, Figure S1: FTIR spectra of undoped PBI and PBI doped with PA, phytic acid, and HPW membranes; Figure S2: Proton conductivity of the membranes along the four consecutive measurement cycles; Figure S3: Bode diagrams for the acid-doped membranes for the first ramp of measurement.
