# Supplementary material for: A Deep Insight into Different Acidic Additives as Doping Agents for Enhancing Proton Conductivity on Polybenzimidazole Membranes
Source: Polymers (Basel). 2020 Jun 18;12(6):1374. doi: 10.3390/polym12061374 (PMC7361977; doi:10.3390/polym12061374)
Supplement: Supplementary file 1 [file polymers-12-01374-s001.pdf]

Supporting Information for

A Deep Insight into Different Acidic Additives as Doping Agents for Enhancing Proton Conductivity on Polybenzimidazole Membranes

Jorge Escorihuela [1,*], Abel García-Bernabé [2] and Vicente Compañ [2,*]

[1] Departamento de Química Orgánica, Facultad de Farmacia, Universitat de València, Av. Vicent Andrés Estellés s/n, 46100 Burjassot, Valencia, Spain
[2] Departamento de Termodinámica Aplicada, Escuela Técnica Superior de Ingeniería Industrial, Universitat Politècnica de València, Camino de Vera s/n, 46022 Valencia, Spain
* Correspondence: jorge.escorihuela@uv.es (J.E.); vicommo@ter.upv.es (V.C.); Tel.: +34-96-387-9328 (V.C.)

Content: **page**

Figure S1. FTIR spectra of undoped PBI and PBI doped with PA, phytic acid, and HPW membranes. S2

Figure S2. Proton conductivity of the membranes along the four consecutive measurement cycles. S2

Figure S3. Long-term conductivity stability of the membranes determined at 25 °C. S3

Figure S4. Bode diagrams for the acid-doped membranes for the first ramp of measurement. S3

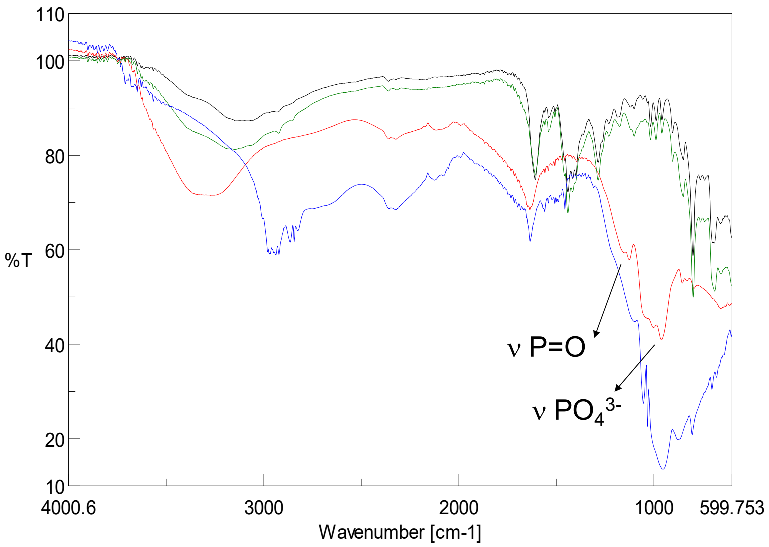

Figure S1. FTIR spectra of undoped PBI (black) and PBI doped with PA (blue), phytic acid (red), and HPW (green) membranes.

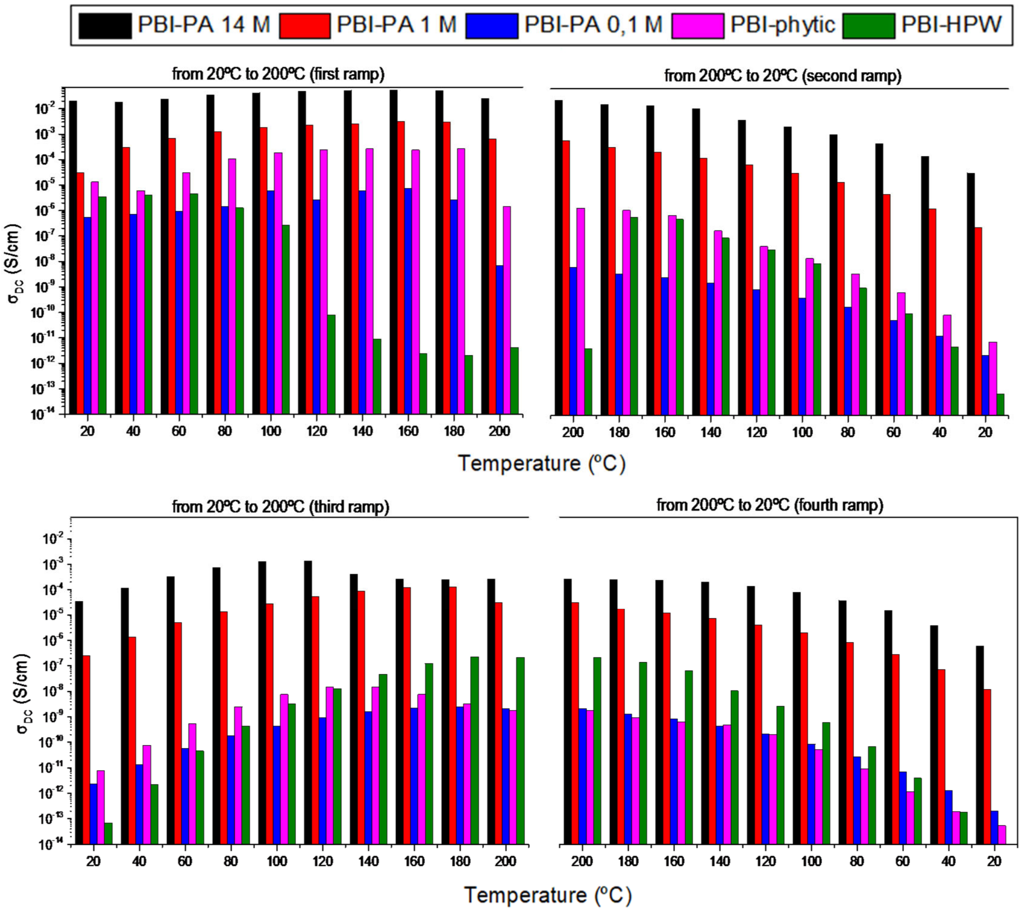

Figure S2. Proton conductivity of the membranes along the four consecutive measurement cycles.

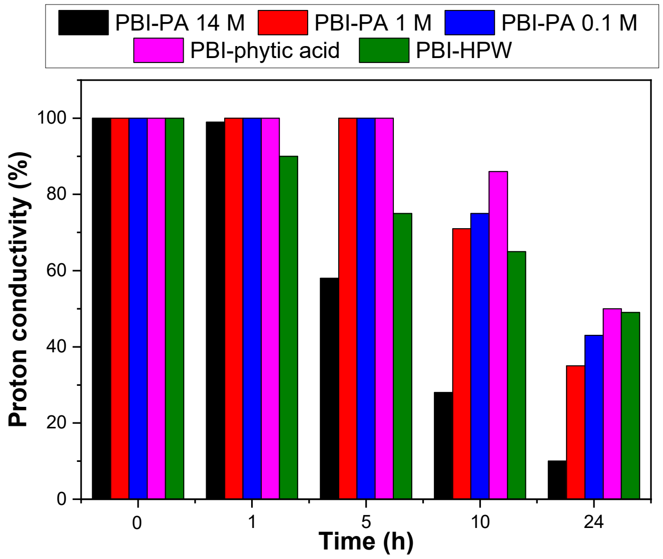

Figure S3. Long-term conductivity stability of the membranes determined at 25 °C.

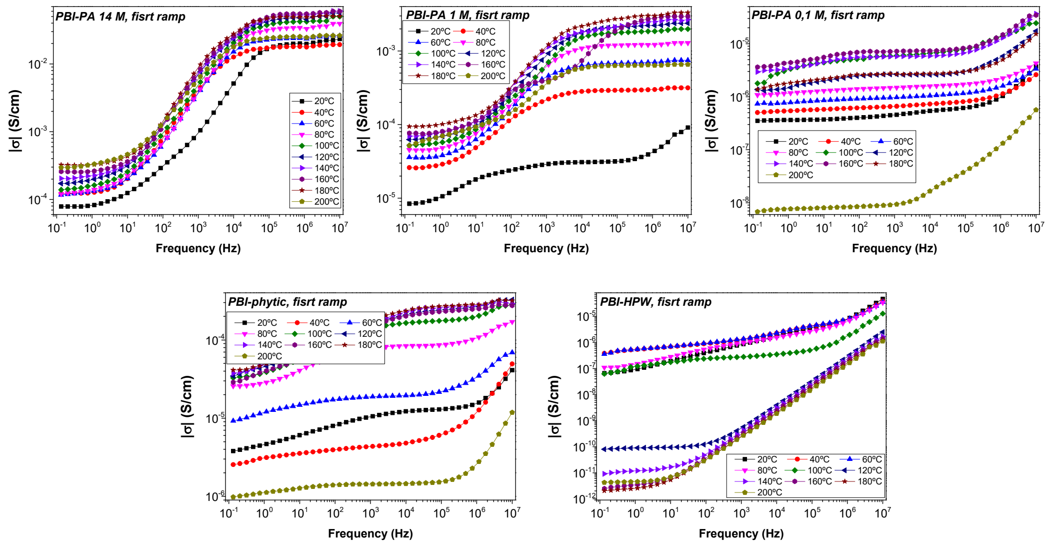

Figure S4. Bode diagrams for the acid-doped membranes for the first ramp of measurement.
